# Effects of far-infrared sauna bathing on recovery from strength and endurance training sessions in men

**DOI:** 10.1186/s40064-015-1093-5

**Published:** 2015-07-07

**Authors:** Antti Mero, Jaakko Tornberg, Mari Mäntykoski, Risto Puurtinen

**Affiliations:** Department of Biology of Physical Activity, University of Jyväskylä, P.O. Box 35, 40351 Jyväskylä, Finland

**Keywords:** Far-infrared, Sauna, Exercise, Neuromuscular, Performance

## Abstract

**Purpose:**

This study investigated effects of far-infrared sauna (FIRS) bathing on recovery from strength training and endurance training sessions, but also possible differences between FIRS and traditional (TRAD) Finnish sauna bathing.

**Methods:**

Ten healthy physically active male volunteers had on various days either a 60 min hypertrophic strength training session (STS) or a 34–40 min maximal endurance training session (ETS), which was following by 30 min bathing in special FIRS sauna at temperature of 35–50°C and humidity of 25–35%. After the sauna, subjects sat for 30 min at room temperature (21°C and 25–30% humidity). In comparison, 30 min of TRAD took place at 35–50°C and in 60–70% humidity. Performance tests included maximal isometric bench press and leg press, counter movement jump (CMJ) and maximal oxygen uptake on a treadmill.

**Results:**

After STS, there were decreases in maximal isometric bench press (p < 0.001), maximal isometric leg press (p < 0.001), CMJ (p < 0.001) and pH (p < 0.001), but increases in heart rate (p < 0.001) and lactate concentration (p < 0.001) as expected. During recovery there were no differences in any variables between FIRS and no sauna bathing (NO SAUNA). Maximal ETS increased oxygen uptake (p < 0.001), heart rate (p < 0.001), lactate concentration (p < 0.001) and decreased pH (p < 0.001) as expected. During recovery at 30 min, CMJ was significantly (p < 0.05) higher (0.34 ± 0.09 m) after FIRS bathing than after sitting with NO SAUNA (0.32 ± 0.0 m). After sauna heart rate was higher (p < 0.05) in TRAD (92 ± 13 beats/min) than in FIRS (71 ± 7 beats/min).

**Conclusion:**

In conclusion, deep penetration of infrared heat (approximately 3–4 cm into fat tissue and neuromuscular system) with mild temperature (35–50°C), and light humidity (25–35%) during FIRS bathing appears favorable for the neuromuscular system to recover from maximal endurance performance. FIRS bathing is a very light loading for the body and provides a comfortable and relaxing experience.

## Background

Sauna bathing (Finnish sauna bathing) has been extensively studied. It is a type of heat exposure, which induces haemodynamic and endocrinological changes in some ways similar to those evoked by physical exercise (e.g. Hannuksela and Ellahham 2001; Kukkonen-Harjula and Kauppinen 2006). In traditional saunas there are either wood stoves or electric heaters to heat the air to approximately 70–100°C, optimally between 80 and 90°C at the face level of the bathers (Kukkonen-Harjula and Kauppinen 2006). The air typically has a relative humidity of 10–20% (Leppäluoto 1988; Kauppinen 1997). The sauna bath consists of repeated cycles of exposure to heat. The length of stay in the sauna depends on each bather’s own sensations of comfort, but the duration usually ranges from 5 to 20 min. This is followed by a cooling-off period (shower, swim, or a period at room temperature), the length of which also depends on personal preference. A sufficient recovery period (usually about 30 min) following a few hot/cold cycles allows for normalizing the body temperature and cessation of sweating.

Some people find the aforementioned practice uncomfortable. In contrast, far-infrared saunas (FIRS) heat to 40–60°C and provide a more comfortable and relaxing experience (Beever 2009). These saunas utilize 120-V infrared elements, similar to the infrared warmers on neonatal resuscitation beds, to radiate heat with a wavelength of around 10 µm. As infrared heat penetrates more deeply (approximately 3–4 cm into fat tissue and the neuromuscular system) than warmed air (only a few millimeters), users develop a more vigorous sweat at a lower temperature than they would in traditional saunas. Consequently, the cardiovascular demand imparted by thermoregulatory homeostasis (sweating, vasodilation, decreased afterload, increased heart rate, and increased cardiac output) is aerobically very light (Beever 2009).

In athletes, the traditional sauna has some positive effects on thermoregulation, if the competition is in a hot environment (Tyka et al. 2008). Also, during weight-reduction, sauna bathing has been used successfully (Viitasalo et al. 1987; Karila et al. 2008). In the recovery from physical exercise, sauna bathing has been used despite it seems that some other methods such as light aerobic exercise, nutrition, massage, sleep, rest are more efficient (e.g. Bompa and Haff 2009). The role of traditional sauna bathing has been more recreational and relaxing in nature. The warm temperatures and cooling-off periods may relax muscles, nerves and blood vessels. This can produce a sensation of calm and relaxation in some people. Conversely, the role of FIRS on recovery has not been scientifically studied. Therefore, the first aim of the present study was to investigate effects of FIRS bathing on recovery from strength training and endurance training sessions. The rationale is primarily based on the fact that the infrared heat penetrates very deeply (approx. 3–4 cm) into the neuromuscular system. This may have positive effects on the neuromuscular system during recovery. The second aim was to study possible differences between FIRS and traditional (TRAD) Finnish sauna bathing.

## Methods

### Subjects

The subjects were 10 healthy male volunteers, who were accustomed to weekly sauna baths. All subjects were physical education students and were involved in recreational physical activity during the previous months. Before the actual experiments, their health-status was checked by a health questionnaire. The subjects had a mean age of 25.3 ± 8.4 years, mean height of 1.78 ± 0.07 m, mean body mass of 79.6 ± 7.5 kg, mean hemoglobin of 160 ± 10 g/l, and mean hematocrit of 0.46 ± 0.02.

### Experimental procedure

The subjects participated in six sauna bath experiments each one separated by 1 week. On the morning, at 08–09 AM, of the experimental sauna bath days (in March–April), the subjects arrived to the lab after 10–12 h fasting. First a fasting blood sample was obtained (basic health parameters not shown here) and then they had a light breakfast. At least 1 h after eating all sauna measurements were performed between 09–12 AM. No food, drink (except 5 dl water in the sauna) or smoking was allowed until the end of the experiment. No other own sauna baths were allowed during the whole study period. Furthermore, strenuous exercise was not allowed for 3 days and alcohol for 2 days before the experimental sessions.

**Figure 1:**
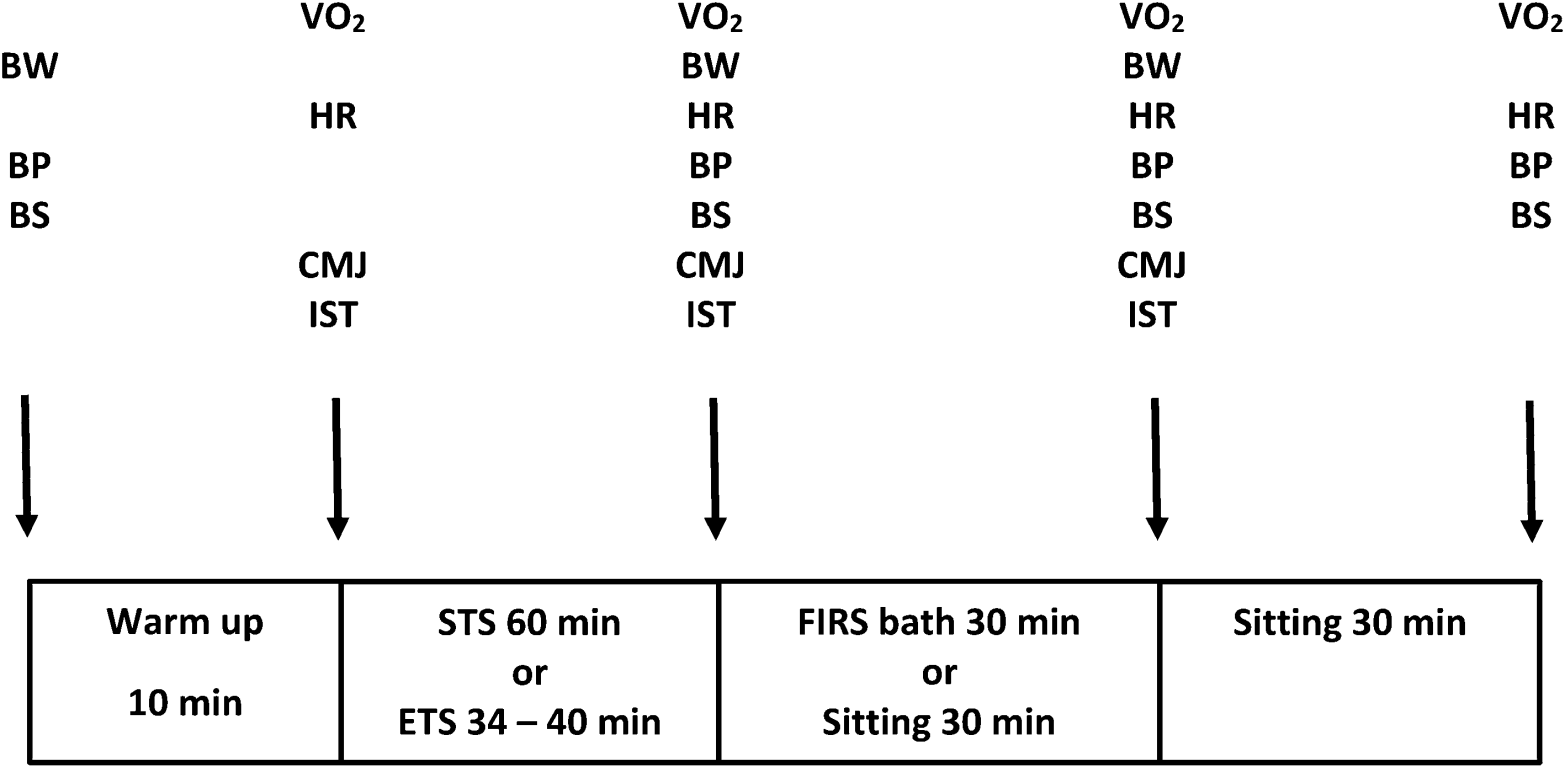
Experimental design. *BW* body weight, *BP* blood pressure, *BS* blood sample, *VO*_*2*_ oxygen uptake, *HR* heart rate, *CMJ* counter movement jump, *IST* isometric strength test, *STS* strength training session, *ETS* endurance training session, *FIRS* far infrared sauna.

In the first experiment, the subjects had 30 min FIRS bathing and then were sat for 30 min at room temperature of 21°C and humidity of 25–30%. Thereafter, the subjects were familiarized with treadmill running, counter movement jump (CMJ), and their one repetition maximum (1RM) in bench press and in bilateral leg press were measured. The next four experiments (strength training session plus FIRS 30 min, strength training session plus no sauna 30 min, endurance training session plus FIRS 30 min, endurance training session plus no sauna 30 min; Figure 1) were performed in a randomized order. At the end of each experiment, the subjects sat for 30 min at room temperature. In the last experiment the subjects had 30 min traditional sauna bathing (TRAD) and then sat for 30 min at room temperature.

### FIRS and TRAD sauna bath

Far-infrared sauna bathing occurred sitting in a special FIRS sauna (Radiant FIRS SGC1210BR, Harvia Ltd, Muurame, Finland). Its width is 1.20 m, depth 1.05 m, height 1.91 m, and voltage 230-V. The type of radiator is carbon fibre and the emitted wavelength is 4–17 µm. The temperature in FIRS sauna was set at 35–50°C (35°C at the level of legs and 50°C at the bather’s face) and relative humidity of 25–35%, respectively. In TRAD, the electrically heated sauna temperature was similar 35–50°C, but relative humidity was increased to 60–70% by throwing water on the hot rocks of the sauna heater. In both saunas the subjects were sitting wearing only shorts and they had to drink 5 dl water during 30 min bathing.

### Strength training session (STS), isometric strength tests (ISTs), and counter movement jump (CMJ)

A 10 min warm-up consisted both 5 min riding a bicycle with heart rate between 120 and 140 beats/min and 5 min dynamic stretching exercises for whole body. After that, the STS lasted 60 min including dynamic hypertrophic training in bilateral bench press and in bilateral leg press and also IST performances in bench press and in leg press. ISTs were performed before and after the training session, as well as following 30 min of FIRS bath or sitting. The bilateral leg IST was performed on an electromechanical dynamometer (David 210, David Health Solutions Ltd., Helsinki, Finland) using three single maximal trials with 2 min recovery between each test. Similarly the maximal bench press performance was measured on an electromechanical dynamometer (Bench Press, Department of Biology of Physical Activity, Jyväskylä, Finland). The arm angle was 90° in bench press and knee angle was 110° in leg press. Maximal CMJ was performed on a contact mat (Newtest Ltd., Oulu, Finland) three times at each measurement point with a recovery of 3 min. The vertical rise of center of gravity was calculated from flight time (Komi and Bosco 1978). The best result out of three trials was selected in CMJ and in ISTs.

The dynamic bench press and dynamic leg press training included 5 × 10RM with a load, which was evaluated according to the 1RM test in pretest. If the subject could not succeed in the last repetitions in a set the researcher assisted slightly to enable the subject to complete all 10 reps. Recovery was 2 min between sets. Each subject carried out the STS at the same time in the morning in both STS experiments and used similar absolute weights in the dynamic training.

### Endurance training session (ETS), VO_2_ and heart rate

A 10 min warm-up consisted both 5 min riding a bicycle with heart rate between 120 and 140 beats/min and 5 min dynamic stretching exercises for whole body. After that, the ETS lasted 34–40 min including 10 min light aerobic work on a bicycle and then running on a treadmill until exhaustion (range 24–30 min). The treadmill exercise began with an 8 km/h speed and 1° angle of the treadmill. Thereafter, the speed was increased by 1 km/h after every 3 min. All subjects were voluntarily exhausted between 24 and 30 min. During running, gaseous exchange was measured using Sensor Medics Breath Gas Analyzer (Vmax series 229, California, USA). The device was calibrated before every measurement and VO_2_ was determined as a mean from the final 30 s of every stage. Heart rate was measured by a Polar heart rate monitor (Polar Electro Oy, Kempele, Finland). The same ISTs and CMJ tests were performed before and after exercise and following 30 min of FIRS bath or sitting.

### Blood pressure

Brachial systolic and diastolic blood pressures were measured from the arm with an electronic blood pressure monitor (Omron M1, Normomedical Ltd, Helsinki, Finland).

### Blood collection and analysis

Blood samples were drawn from the antecubital vein in a sitting position. Analysis included hemoglobin (Hb), serum total testosterone, cortisol, growth hormone, lactate, and pH. Serum samples were kept frozen at −80°C until analyzed. Two milliliters of blood were taken in K2 EDTA tubes (Terumo Medical Co., Leuven, Belgium) for measurements of Hb concentration with a Sysmex KX 21N Analyzer (Sysmex Co., Kobe, Japan). For the determination of serum hormone concentrations, five milliliters of blood were taken into serum separator tubes and the concentrations were analyzed by an immunometric chemiluminescence method with Immulite^®^ 1000 (DPC, Los Angeles, USA). The sensitivities of the assays were 0.5 nmol/l for testosterone, 5.5 nmol/l for cortisol, and 2.6 µg/l for growth hormone. The intra-assay coefficient of variation (CV) was 5.7% for testosterone, 4.6% for cortisol, and 4.2% for growth hormone. Blood samples for lactate were obtained from the fingertip and collected into capillary tubes (20 µl), which were placed in a 1 ml hemolyzing solution and analysed automatically after the completion of testing according to the manufacturer’s instructions (EKF diagnostic, C-line system, Biosen, Germany). pH was analyzed with IL GEM Premier 3000 Blood Gas System (Instrumentation Laboratory, Lexington, MA, USA). The intra-assay CV was 0.1% for pH.

### Statistics

Before applying statistical methods, the data was checked for normality by Shapiro–Wilk’s test and the homogeneity of variances by Levene’s test. Then statistical analyses were performed with PAWS Statistics version 20.0 for Windows (SPSS, Inc, Chicago, IL, USA). Differences between conditions were determined through one-way ANOVA. Bonferroni correction was used as a post hoc test. Data are presented as mean ± SD. The statistical difference was considered to be significant at the p < 0.05 level.

## Results

### FIRS and TRAD

Table 1 presents the results for FIRS and TRAD. After sauna, heart rate was higher (p < 0.05) in TRAD (92 ± 13 beats/min) than in FIRS (71 ± 7 beats/min). Serum cortisol decreased in both FIRS and in TRAD being at the lowest (p < 0.05) at the end of recovery. Serum growth hormone increased in both FIRS and in TRAD and was highest (p < 0.05) after sauna and at 30 min of recovery.

**Table 1:** Measured variables in 30 min FIRS bathing and in 30 min traditional (TRAD) sauna bathing (mean ± SD)

Variable	Before sauna	15 min in sauna	After sauna	Recovery 30 min
Body mass (kg)^a^				
FIRS	79.7 ± 7.1		79.4 ± 7.5	
TRAD	80.6 ± 8.6		80.1 ± 8.6	
Hemoglobin (g/l)				
FIRS	159 ± 10	157 ± 10	157 ± 9	156 ± 9
TRAD	158 ± 8	160 ± 8	160 ± 9	159 ± 8
Heart rate (beats/min)				
FIRS	70 ± 9	72 ± 9	71 ± 7	64 ± 7
TRAD	71 ± 8	81 ± 10	92 ± 13^c^	68 ± 9
Blood pressure (mm Hg)				
FIRS systolic	138 ± 13	127 ± 14	129 ± 11	129 ± 12
FIRS diastolic	83 ± 15	73 ± 13	77 ± 11	78 ± 10
TRAD systolic	133 ± 13	129 ± 14	133 ± 11	127 ± 12
TRAD diastolic	76 ± 15	74 ± 13	75 ± 11	82 ± 10
pH				
FIRS	7.40 ± 0.03	7.42 ± 0.02	7.42 ± 0.02	7.40 ± 0.02
TRAD	7.41 ± 0.02	7.43 ± 0.02	7.43 ± 0.03	7.42 ± 0.02
Testosterone (nmol/l)				
FIRS	18.8 ± 4.7	18.3 ± 5.3	17.9 ± 5.1	18.9 ± 6.3
TRAD	20.0 ± 5.3	18.7 ± 3.5	21.0 ± 5.6	19.8 ± 5.5
Cortisol (nmol/l)				
FIRS	399 ± 88	367 ± 122	354 ± 133	329 ± 109^b^
TRAD	546 ± 122	467 ± 143^b^	409 ± 117^b^	380 ± 127^b^
Growth hormone (µg/l)				
FIRS	0.7 ± 1.3	5.2 ± 12.6	5.4 ± 5.1^b^	11.2 ± 8.0^b^
TRAD	0.6 ± 0.6	6.9 ± 8.7	19.4 ± 26.1^b^	11.9 ± 20.0^b^

^a^Subjects drank 0.5 dl water in sauna which is included in body mass.

^b^Significantly (p < 0.05) different from before value.

^c^Significantly (p < 0.05) different from FIRS after sauna value.

### STS, FIRS and recovery sitting

The average training session work load of the subjects was in bench press 2,505 ± 346 kg and in leg press 7,583 ± 998 kg. Maximal isometric bench press, maximal isometric leg press and CMJ decreased significantly (p < 0.001) after STS, but there were no differences between FIRS and NO SAUNA (Table 2). Also, in the other variables there were expected but similar changes after STS in the groups.

**Table 2:** Measured variables in STS and FIRS bathing 30 min or in STS and sitting in a normal room (NO SAUNA) 30 min and in both cases during recovery sitting another 30 min (mean ± SD)

Variable	Before STS	After STS	Recovery 30 min	Recovery 60 min
Isometric bench press (kg)				
FIRS	96.6 ± 14.6	78.9 ± 12.4^a^	83.7 ± 13.2^a^	–
NO SAUNA	97.2 ± 15.9	80.3 ± 12.7^a^	84.6 ± 13.1^a^	–
Isometric leg press (kg)				
FIRS	428 ± 79	374 ± 70^a^	383 ± 78^a^	–
NO SAUNA	424 ± 70	362 ± 69^a^	372 ± 83^a^	–
Heart rate (beats/min)				
FIRS	68 ± 10	129 ± 16^a^	87 ± 10	77 ± 10
NO SAUNA	67 ± 8	125 ± 8^a^	76 ± 8	67 ± 8
Blood pressure (mm Hg)				
FIRS systolic	130 ± 13	158 ± 15^a^	129 ± 13	128 ± 12
FIRS diastolic	74 ± 9	75 ± 18	76 ± 6	78 ± 12
NO SAUNA systolic	132 ± 15	163 ± 13^a^	129 ± 10	129 ± 12
NO SAUNA diastolic	74 ± 9	75 ± 18	76 ± 6	78 ± 12
pH				
FIRS	7.41 ± 0.02	7.32 ± 0.06^a^	7.42 ± 0.02	7.41 ± 0.02
NO SAUNA	7.41 ± 0.02	7.34 ± 0.02^a^	7.40 ± 0.03	7.41 ± 0.02
Lactate (mmol/l)				
FIRS	1.8 ± 0.9	13.2 ± 1.7^a^	4.4 ± 1.6	2.4 ± 0.9
NO SAUNA	2.6 ± 1.7	12.1 ± 1.9^a^	3.8 ± 0.8	2.5 ± 0.7
Testosterone (nmol/l)				
FIRS	18.1 ± 5.2	22.7 ± 6.5^b^	20.3 ± 5.4	20.1 ± 5.9
NO SAUNA	18.5 ± 7.5	22.3 ± 6.4^b^	20.5 ± 7.5	19.8 ± 7.4
Cortisol (nmol/l)				
FIRS	471 ± 162	449 ± 159	435 ± 213	368 ± 242^b^
NO SAUNA	467 ± 197	441 ± 216	401 ± 219	320 ± 189^b^
Growth hormone (µg/l)				
FIRS	0.2 ± .0.2	43.3 ± 36.6^a^	16.6 ± 12.2^b^	6.5 ± 7.0
NO SAUNA	1.3 ± 3.5	40.5 ± 41.9^a^	13.5 ± 17.3^b^	6.6 ± 8.8
Body mass (kg)				
FIRS	81.1 ± 6.3	80.8 ± 6.2	80.7 ± 6.2^c^	80.6 ± 6.2^c^
NO SAUNA	80.9 ± 6.6	80.6 ± 6.6	80.5 ± 6.6	80.5 ± 6.6
CMJ (m)				
FIRS	0.37 ± 0.05	0.33 ± 0.05^a^	0.34 ± 0.05^a^	–
NO SAUNA	0.37 ± 0.06	0.33 ± 0.07^a^	0.33 ± 0.08^a^	

^a^Significantly (p < 0.001) different from before value.

^b^Significantly (p < 0.05) different from before value.

^c^Subjects drank 0.5 dl water in sauna which is included in body mass.

### ETS, FIRS and recovery sitting

Table 3 presents the results for ETS and FIRS 30 min bathing or for ETS and sitting in a normal room (NO SAUNA) 30 min and, in both cases, during recovery sitting another 30 min. Maximal ETS increased heart rate (p < 0.001), lactate concentration (p < 0.001) and decreased pH (p < 0.001) as expected in both groups. CMJ after FIRS bathing was significantly (p < 0.05) higher (0.34 ± 0.09 m) than after sitting with no sauna (0.32 ± 0.0 m).

**Table 3:** Measured variables in ETS and FIRS bathing 30 min or in ETS and sitting in a normal room (NO SAUNA) 30 min and in both cases during recovery sitting another 30 min (mean ± SD)

Variable	Before ETS	After ETS	Recovery 30 min	Recovery 60 min
VO_2_ (ml/kg/min)				
FIRS	5.7 ± 0.9	62.7 ± 4.2^a^	5.3 ± 0.8	4.9 ± 0.6
NO SAUNA	5.7 ± 0.7	62.9 ± 3.5^a^	5.4 ± 0.9	5.0 ± 0.6
Heart rate (beats/min)				
FIRS	75 ± 9	201 ± 11^a^	99 ± 9	80 ± 11
NO SAUNA	75 ± 10	200 ± 10^a^	93 ± 11	79 ± 14
Blood pressure (mm Hg)				
FIRS systolic	139 ± 12	168 ± 20^b^	114 ± 11	121 ± 8
FIRS diastolic	80 ± 10	80 ± 12	73 ± 10	72 ± 6
NO SAUNA systolic	138 ± 12	182 ± 17^b^	125 ± 12	126 ± 8
NO SAUNA diastolic	78 ± 10	86 ± 17	74 ± 10	75 ± 6
pH				
FIRS	7.40 ± 0.0	7.30 ± 0.04^a^	7.40 ± 0.02	7.40 ± 0.02
NO SAUNA	7.41 ± 0.02	7.29 ± 0.04^a^	7.40 ± 0.03	7.41 ± 0.02
Lactate (mmol/l)				
FIRS	2.0 ± 1.0	12.0 ± 2.7^a^	4.4 ± 2.3	2.1 ± 0.4
NO SAUNA	1.8 ± 0.6	12.9 ± 4.1^a^	4.4 ± 2.1	2.3 ± 0.8
Testosterone (nmol/l)				
FIRS	18.8 ± 3.9	23.6 ± 5.1	20.6 ± 5.1	20.4 ± 5.5
NO SAUNA	18.5 ± 4.4	23.5 ± 6.4	19.5 ± 6.7	18.0 ± 4.7
Cortisol (nmol/l)				
FIRS	531 ± 151	640 ± 132	601 ± 121	491 ± 132
NO SAUNA	519 ± 117	639 ± 112	616 ± 196	609 ± 211
Growth hormone (µg/l)				
FIRS	2.2 ± .0.9	65.5 ± 41.6^a^	26.6 ± 11.2^b^	7.5 ± 8.0
NO SAUNA	1.5 ± 3.8	57.5 ± 39.9^a^	23.5 ± 16.3^b^	7.6 ± 8.8
Body mass (kg)				
FIRS	79.8 ± 7.6	79.4 ± 7.4	78.9 ± 7.4^c^	78.9 ± 7.4^c^
NO SAUNA	79.6 ± 7.6	79.1 ± 7.5	78.9 ± 7.5	78.9 ± 7.4
CMJ (m)				
FIRS	0.37 ± 0.08	0.32 ± 0.10	0.34 ± 0.09^d^	–
NO SAUNA	0.37 ± 0.08	0.32 ± 0.09	0.32 ± 0.08	–

^a^Significantly (p < 0.001) different from before value.

^b^Significantly (p < 0.05) different from before value.

^c^Subjects drank 0.5 dl water in sauna which is included in body mass.

^d^Significantly (p < 0.05) different from NO SAUNA value.

## Discussion

### Main findings

The main results showed that maximal isometric bench press, maximal isometric leg press and CMJ decreased strongly after STS as expected, but during recovery there were no differences in any variables between FIRS and NO SAUNA bathing. Maximal ETS increased heart rate, blood lactate concentration and decreased pH as expected. During recovery from ETS at 30 min CMJ was significantly higher after FIRS bathing than after sitting with NO SAUNA bathing. Without training sessions, heart rate was higher after TRAD sauna than after FIRS bathing.

### FIRS, STS and ETS

Maximal isometric strength both in legs and arms and CMJ decreased strongly after the hypertrophic type STS as expected and were not recovered after 30 min either in FIRS or in NO SAUNA condition. Also, as expected, there were large increases in heart rate, blood pressure, acidity, lactate, testosterone and growth hormone concentrations immediately after STS. All these variables were recovered at 30 min after STS except growth hormone, which recovered after 1 h. It was surprising to see a non-significant decrease in the cortisol concentration immediately after STS and at 30 min during recovery. One hour after STS the decrease was already strong. As discussed earlier, the circadian rhythm tends to strongly decrease cortisol in the morning, whereas a resistance training stimulus tends to increase an acute cortisol concentration (e.g. Kraemer and Ratamess 2005). Obviously, the overall stress of STS was not sufficient to increase the concentration in the morning. Overall, during recovery after STS there were no differences in any variables between FIRS and NO SAUNA bathing. The only trend difference (p = 0.11) between FIRS and NO SAUNA conditions was observed in CMJ, where performance was slightly better at 30 min recovery in FIRS bathing.

Oxygen uptake increased, probably to its maximum, at the end of the 34–40 min ETS. This is supported by the high heart rate values. Also, blood pressure, acidity, and lactate increased strongly at the end of ETS. All those variables recovered during 30 min and there were no differences between FIRS and NO SAUNA bathing. There were increases in the concentrations of all three hormones, but only statistically significant in growth hormone. GH concentration recovered slowly and returned to baseline 1 h after ETS. The profile of cortisol was different from STS, because it increased at the end of ETS, although the increase was not significant. Consequently, it seems that ETS was metabolically slightly more stressful than STS, especially when heart rate was 200 beats/min. Recovery of the legs from ETS was better with FIRS bathing, because CMJ was clearly better compared to NO SAUNA condition after 30 min. The reason for greater recovery in jumping ability may be infrared heat during 30 min penetrating deeply (approx. 3–4 cm) into fat tissue and the neuromuscular system (Beever 2009) compared to the air at room temperature 21°C and humidity 25–30% in NO SAUNA condition. Consequently, the heated force production and relaxation of the leg muscles were better. This result is partly confirmed with the result in the STS condition, although it was only a trend.

### FIRS and TRAD sauna bathing

In TRAD saunas there are either wood stoves or 220-V heaters (used in the present study) to heat air to 70–100°C, which then heats the bather mainly via convection. The air has a relative humidity of 10–20% (Leppäluoto 1988; Kauppinen 1997). It is typical of a Finnish sauna to have dry air and a high temperature (Karjanoja et al. 1997). However, many bathers in Finland throw water on the hot rocks of the sauna heater. Consequently, humidity increases strongly even up to 80%. FIRSs heat air to 50–60°C providing a more comfortable and relaxing experience (Beever 2009). As infrared heat penetrates more deeply into fat and the neuromuscular system than warmed air, bathers develop a more vigorous sweat at a lower temperature than they would in TRAD saunas (Beever 2009). In the present study, we wanted to compare FIRS bathing to TRAD sauna bathing in a control condition using the same temperature of 35–50°C, but in TRAD sauna the relative humidity was increased to 60–70% (compared to 25–35% in FIRS) by throwing water on the hot rocks of the sauna heater. This was done, because most of the Finnish athletes and recreational people use this type of TRAD sauna bathing during recovery from intensive exercise.

Heart rate was 92 beats/min immediately after TRAD sauna bathing, which was higher than after FIRS sauna bathing (71 beats/min). The mean value of all three measurement points during recovery was 16% higher after TRAD sauna bathing. The main reason for the results is the large difference in relative humidity. There were no differences in other measured variables between the two experiments. The concentration of testosterone was unchanged after both saunas, which confirms earlier studies (Leppäluoto et al. 1986; Kukkonen-Harjula et al. 1989). It is interesting to observe decreases in cortisol concentrations during recovery from these two light “aerobic” sauna conditions, which may partly be due to the early morning time between 09–12 AM. It is known that normally, the highest cortisol secretion occurs in the second half of the night with peak cortisol production occurring in the early morning. Following this, cortisol concentration declines throughout the day with the lowest concentrations late in the evening (e.g. Tsigos and Chrousos 2002). Results in earlier studies investigating sauna bathing and cortisol are somewhat conflicting (Hannuksela and Ellahham 2001). Growth hormone concentration increased significantly also in both sauna conditions, which confirms earlier studies (Kukkonen-Harjula et al. 1989; Hannuksela and Ellahham 2001; Pich et al. 2003). Finally, the results with FIRS bathing show that it is a very light loading for the body and provides a comfortable and relaxing experience.

## Conclusion

In conclusion, deep penetration of infrared heat (approximately 3–4 cm into fat tissue and neuromuscular system) under mild temperature (35–50°C), and light humidity (25–35%) conditions during FIRS bathing are favorable for the neuromuscular system to recover from maximal endurance performance. In practice, FIRS bathing may be used among other recovery methods in athletes and also in other physically active people. FIRS bathing is a very light loading for the body compared to TRAD and provides a comfortable and relaxing experience.

## Abbreviations

FIRS: far-infrared sauna
STS: strength training session
ETS: endurance training session
TRAD: traditional
CMJ: counter movement jump
BW: body weight
BP: blood pressure
BS: blood sample
VO_2_: oxygen uptake
HR: heart rate
IST: isometric strength test
